# Exploration of risk factors for the incidence of knee osteoarthritis in rural areas of northern China and the establishment of a prediction model

**DOI:** 10.1371/journal.pone.0338003

**Published:** 2025-12-29

**Authors:** Junhui Ma, Qiang Ma, Chao Shi, Bing Zhuan, Jun Ma

**Affiliations:** 1 Department of Respiratory and Critical Care Medicine, People’s Hospital of Ningxia Hui Autonomous, Yinchuan, China; 2 Thoracic Surgery Department, People's Hospital of Ningxia Hui Autonomous, Yinchuan, China; 3 Department of Central Labororatory, People’s Hospital of Ningxia Hui Autonomous Region, Yinchuan, China; 4 Department of Sports Medicine, People’s Hospital of Ningxia Hui Autonomous Region, Yinchuan City, Ningxia, China; MountainView Hospital, UNITED STATES OF AMERICA

## Abstract

**Objective:**

This study sought to identify knee osteoarthritis (KOA) contributing factors and develop a preliminary forecasting model for its development.

**Methods:**

Participants were systematically invited to complete an exhaustive medical questionnaire designed to capture relevant health and demographic information. Following data collection, univariate analyses were conducted to assess the significance of the variables obtained from the questionnaire. To delineate the association between identified risk factors and the occurrence of KOA, a binary logistic regression model was utilized. The reliability of the model was evaluated through internal validation, encompassing both calibration and discrimination analyses. Calibration was quantified using the Hosmer–Lemeshow χ² statistic to assess the model’s goodness of fit, while discrimination was gauged utilizing the receiver operating characteristic (ROC) curve, providing a comprehensive evaluation of the model’s predictive accuracy.

**Results:**

In the present study, a total of 445 cases were analyzed, with 266 cases employed for model development and 179 cases reserved for internal validation. Univariate analysis revealed significant statistical differences between the two groups with respect to several variables, including family history of KOA, heating methods, stair usage, anxiety and depression, toilet type, and the frequency of consumption of vegetables, fruits, red meat, and dairy products. Binary logistic regression analysis identified advanced age, lower educational level, use of a squat toilet, family history of KOA, and psychological conditions such as anxiety and depression as significant risk factors for the development of KOA. Furthermore, a moderate predictive value was observed for incident KOA based on a combination of factors, including age, gender, weight, height, family history of KOA, toilet type, mode of transportation, dairy product consumption, and emotional state.

**Conclusions:**

Our findings indicate that, in addition to established risk factors such as age, gender, height, and weight, lifestyle and dietary habits also play a pivotal role in the etiology of KOA. These factors not only serve as potential risk markers but also exhibit predictive utility for the onset of KOA, suggesting a comprehensive approach to prevention and intervention strategies.

## Introduction

Osteoarthritis (OA), recognized as the most prevalent joint disorder globally [1], ranks as a significant contributor to disability and is among the most common chronic illnesses [2]. Characterized by pivotal pathological features such as cartilage degeneration, osteophyte formation, and subchondral bone sclerosis, OA results in chronic pain, functional impairment, and a diminished quality of life for affected individuals [3,4]. Therapeutic interventions, including arthroplasty and osteotomy, although effective, often entail substantial financial costs, thereby exacerbating the economic burden on OA patients [5].

KOA, a prevalent chronic joint disorder, ranks as the predominant cause of lower limb disability among the elderly population [6]. In the pursuit of preventive strategies, it is imperative to delineate the risk factors contributing to this condition. These factors can be categorized into two distinct groups: nonmodifiable risks and potentially modifiable risks. Nonmodifiable risks encompass intrinsic attributes such as age, gender [7], genetic susceptibility, and family history. Conversely, potentially modifiable risks include body mass index (BMI), occupational hazards [8], joint injury, quadriceps weakness, nutrient deficiencies, bone mineral density, and oestrogen insufficiency [9,10].

To date, a plethora of predictive models have been developed to estimate the incidence of KOA. Among these, the model proposed by H.J. M. Kerkhof [11] stands out as the most robustly supported by empirical evidence. This model, which was derived from three distinct populations, reveals that the inclusion of readily accessible ‘Questionnaire’ variables, genetic markers, OA at other joint sites, and biochemical markers contributes only marginally to the predictive accuracy of KOA incidence when compared to the traditional parameters of age, gender, and body mass index (BMI) within an elderly cohort.

Nevertheless, the paucity of empirical research investigating the impact of dietary habits and lifestyle on KOA in the rural regions of northern China remains a notable gap in the current literature. Against this backdrop, the primary objective of our study was to delineate the risk factors associated with KOA utilizing a questionnaire-based survey approach, ultimately aiming to construct a robust risk prediction model.

## Materials and methods

### Study design and setting

This investigation was designed as a cross-sectional study, utilizing a comprehensive questionnaire (S1 File) and radiographic assessment of the knees. The questionnaire, which encompassed basic demographic information, lifestyle factors, dietary habits, and transportation modes, was administered to all participants by a team of skilled interviewers. Each participant underwent a complimentary radiographic examination of both knees to facilitate the collection of detailed imaging data. In May 2022, we conducted information collection in several randomly selected rural areas.

All participants granted oral informed consent, and we exclusively retrieved the survey instruments and conducted knee X-ray examinations on those who acceded. This study does not involve minors. This study has been reviewed and approved by the Ethics Committee of Ningxia Hui Autonomous Region People’s Hospital(Ethics Approval Number:[2021]-YCSKY-010).

### Participants

In the context of our study, the following inclusion and exclusion criteria were meticulously established to ensure the integrity and relevance of the research findings:

Inclusion Criteria:(1) Participants must be aged 40 years or older. (2) Gender is not a limiting factor, and individuals of any gender are eligible. (3) Participants must possess the cognitive capacity to comprehend the investigation procedure, provide informed consent by signing a written consent form, and explicitly express their willingness to partake in the study.

Exclusion Criteria:(1)Individuals who are unable to provide informed consent are ineligible.(2)Those with terminal illnesses or mental health disorders are excluded to avoid potential confounding variables.(3) Participants with inflammatory joint diseases are precluded from the study due to the potential impact on the investigation’s outcomes.(4)Individuals diagnosed with dementia are excluded to ensure the validity of the study’s data.(5)Those who have sustained acute soft tissue injuries to the knee within the past week are not eligible to minimize the influence of recent trauma on study results.(6)Participants who have undergone knee replacement surgery are excluded to focus on individuals with intact knee joints.(7) Pregnant and lactating women are excluded to avoid any potential confounding effects related to pregnancy or lactation.(8)Individuals with serious complications that could interfere with the study’s objectives or outcomes are excluded to maintain the homogeneity of the study population.

### The progress of modeling

Data preprocessing: the missing rate of all continuous variables in this study was less than 10%. Based on the principles of data integrity and statistical rationality, multiple linear regression imputation was adopted for missing value handling. Specifically, a regression model incorporating other risk factors (e.g., age, BMI, dietary habits, and psychological status) was constructed, and missing values were predicted using the correlations between variables. Compared with mean/median imputation, this method can more accurately retain the distribution characteristics of data and reduce bias.

Prior to imputation, the distribution differences of continuous variables (e.g., height, weight, and dietary frequency) between the two groups were tested using independent samples t-tests (for normally distributed data) or Mann-Whitney U tests (for non-normally distributed data). Meanwhile, chi-square tests were used to examine the differences in the composition ratios of core categorical variables (e.g., family history and toilet type) between the two groups. The results showed that there were statistically significant differences in the distribution of family history and frequency of dairy product consumption between the case group (KOA group) and the control group (P < 0.05). Therefore, imputation operations were performed separately for the two groups to avoid confusion of data characteristics between groups and ensure that the imputed data better conformed to the actual situation of each group.

The variables with missing values and their corresponding missing rates in this study are as follows: height (1.35%, 6/446), weight (0.89%, 4/446), frequency of vegetable and fruit consumption (1.12%, 5/446), frequency of red meat and its product consumption (0.67%, 3/446), frequency of dairy product consumption (1.57%, 7/446), and anxiety and depression status (0.45%, 2/446). No missing data were observed for other variables, including age, gender, family history, toilet type, and heating method. All missing rates were calculated based on the 446 cases finally included in the analysis.

Dataset Balancing: To address the slight imbalance in sample size between the case group (149 cases) and the control group (118 cases), the Synthetic Minority Oversampling Technique (SMOTE) was used to balance the dataset during the data preprocessing stage prior to model training. Only the 60% model training set (267 cases) was balanced, while the 40% internal validation set (179 cases) retained its original sample distribution to ensure the authenticity of validation results.

Specifically, the control group (minority class) served as the basis. The SMOTE algorithm calculated the feature differences between each minority class sample and its neighboring samples, generating 31 new minority class samples. After balancing, both the case group and the control group in the training set had 149 samples, achieving a 1:1 distribution.

After sample generation, the Kolmogorov-Smirnov test was used to verify the distribution characteristics of key continuous variables (e.g., height, weight), and the chi-square test was used to verify the distribution of categorical variables (e.g., family history, toilet type). The results showed no statistically significant difference in distribution between the synthetic samples and the original minority class samples (P > 0.05), ensuring that no new bias was introduced during the balancing process.

In the initial phase of our analytical approach, we employed univariate statistical techniques to evaluate the association between individual risk factors and the onset of incident KOA. This exploratory analysis provided a foundation for the identification of significant risk factors. Building upon these findings, we subsequently constructed a multivariate logistic regression model, incorporating only those risk factors that were deemed statistically meaningful. This methodological strategy enabled us to derive a robust predictive model that accounts for the complex interplay of risk factors in the context of knee OA development.

Subsequently, this study conducted a rigorous validation of the proposed model, focusing on both calibration and discrimination parameters. Calibration was employed to evaluate the accuracy of the predicted probabilities, utilizing the Hosmer–Lemeshow χ² statistic for goodness of fit. This metric enabled a comparative analysis between the observed and predicted risk deciles, with smaller values denoting optimal calibration.

Discrimination, on the other hand, was assessed to gauge the model’s proficiency in accurately classifying subjects into disparate risk groups. For this purpose, the area under the ROC curve was adopted as a measure of discriminative efficacy. The ROC curve plotting sensitivity against 1-specificity across various risk score cut-off points provided a visual representation of the model’s discriminative capacity, where larger ROC values signified enhanced discriminative power. The diagnostic outcomes for KOA are delineated in Table 1, while the definitions and descriptions of the predictors utilized in the model are provided in Table 2 for clarity and reference.

**Table 1:** Diagnostic criteria for KOA.

Serial number	Symptom or sign
1	Recurring knee pain in the past month
2	Radiographic evidence of KOA, including visualization of the tibiofemoral (weight-bearing posteroanterior view) and patellofemoral (lateral or skyline view) compartments, which may exhibit features such as osteophytes, subchondral sclerosis, cysts, bone attrition, and asymmetric joint-space narrowing
3	Age ≥ 50 years
4	Morning stiffness lasts less than 30 minutes
5	Bone friction or a sensation of bone rubbing during movement

Annotation: The diagnosis of KOA can be established by meeting condition 1, in conjunction with any two of the following conditions (2, 3, 4, or 5).

**Table 2:** Operational Definitions of Predictors.

Predictor	Definition
Method of heating	The prevalent approach employed to maintain warmth during the winter season.
Transportation	The primary mode of conveyance utilized for daily commuting and travel.
Toilet type	Include sit – down toilet and squat toilet.
Walk up and down stairs	The frequency with which individuals traverse stairs, indicating physical activity levels.
Vegetables and fruits	The frequency of consumption of fruits and vegetables in the diet.
Red meat and its products	The frequency of consumption of red meat and its derivatives.
Dairy products	The frequency of consumption of milk and milk-based products.
Anxiety and depression	The psychological state indicating the presence of feelings of depression or anxiety.

### Statistical analysis

All statistical analyses were conducted utilizing SPSS version 25. A p-value of less than 0.05 was deemed statistically significant, whereas a p-value below 0.01 was considered highly significant.

## Results

### Population characteristics

**Fig 1:**
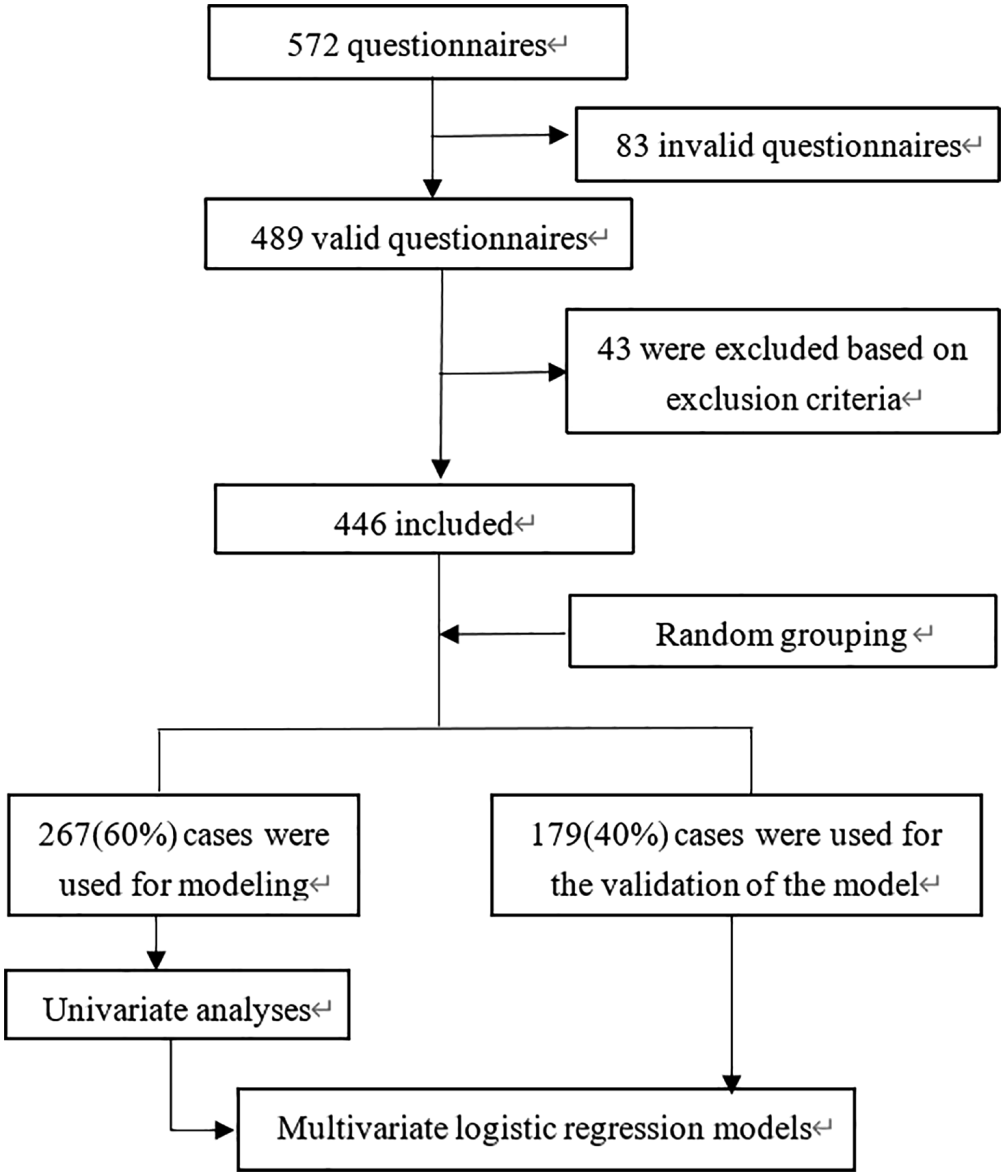
Flowchart of participant inclusion and exclusion. A comprehensive dataset consisting of 572 questionnaires was initially gathered. Upon meticulous review, 83 questionnaires were deemed invalid due to incomplete or inconsistent responses. Subsequently, an additional 43 participants were excluded based on predefined exclusion criteria. Consequently, a total of 446 cases were included in the present research, forming the basis for the subsequent analysis.

A comprehensive dataset comprising 572 questionnaires was meticulously assembled for this study. Upon meticulous screening, 83 questionnaires were identified as invalid, while an additional 43 were excluded based on predefined exclusion criteria. Consequently, a total of 446 cases (S1 Table) were deemed eligible for inclusion in this research. To facilitate subsequent analyses, these cases were randomly allocated into two distinct groups: a training group and a verification group, with allocations of 60% and 40%, respectively. Specifically, 267 cases were utilized for the development of the model, while 179 cases were reserved for the internal validation of the model (Fig 1).

The fundamental demographic characteristics of the KOA cohort and the control group are delineated in Table 3. It is observed that the KOA group comprised 149 incident cases that conformed to the diagnostic criteria outlined in Table 1, whereas the control group consisted of 118 participants. Notably, there were significant differences between the two groups in terms of occupation and educational level, as well as height. In contrast, no statistically significant disparities were found with respect to gender, weight, and age.

**Table 3:** The baseline information of the two groups.

Factors	KOA Group (n = 149)	Control Group (n = 118)	P-value
Gender			0.381
Male/Female	57/92	52/66	
Height(cm)(SD)	162.75(6.8)	164.96(6.8)	0.009
Weight [M (P_25_, P_75_) Kg]	65.0(61.0,72.5)	66.0(61.8,75.0)	0.360
BMI(Kg/m^2^)			<0.05
< 25.0	140(93.96%)	9(7.63%)	
25.0-29.9	7(4.70%)	90(76.27%)	
> 30.0	2(1.34%)	19(16.10%)	
Age(y)			0.092
40-49	15(10.07%)	23(19.49%)	
50-59	41 (27.52%)	37(31.36%)	
60-69	45(30.20%)	28(23.73%)	
＞ 70	48(32.21%)	30(25.42%)	
Educational level			0.002
No diploma	57(38.26%)	28(23.73%)	
Junior diploma	83(55.70%)	69(58.47%)	
High school or above	9(6.04%)	21(17.80%)	
Occupation			0.004
Farmer, fisherman, shepherd	102 (68.46%)	60(50.85%)	
Others	47(31.54%)	58(49.15%)	

Note: SD = Standard Deviation; M = Median; P25, P75 = 25th and 75th percentiles, respectively. BMI = Body Mass Index. Statistical significance was determined using chi-square tests for categorical variables and t-tests for continuous variables.

### The relationship between risk factors and incident KOA

In the present study, we administered a comprehensive questionnaire consisting of 62 questions to our participants. Following univariate analysis, the variables that emerged as significant are delineated in Table 4. Notably, the KOA cohort exhibited a higher prevalence of family history compared to the control group. A marked discrepancy in heating methods was observed between the two groups, with the KOA group demonstrating a higher incidence of burning mineral fuel. Moreover, the KOA group reported a significantly higher frequency of utilizing sit-down toilets, ascending and descending stairs, and experiencing anxiety and depression relative to the control group. Statistically significant differences were also identified in the consumption frequencies of vegetables, fruits, red meat (and its derivatives), and dairy products between the two groups.

**Table 4:** Univariate Analysis of Risk Factors Between KOA Cases and Control Groups.

Factors	KOA Group (n = 149)	Control Group (n = 118)	P-value
Family history of KOA			0.006
Yes/ No	45/104	18/100	
Method of heating			<0.05
No heating	3(2.01%)	10(%)	
Central-heating	89(59.73%)	91(%)	
Burning mineral fuel	57(38.26%)	17(%)	
Transportation			<0.05
Walk	52(34.90%)	69(%)	
Bike	17(11.41%)	12(%)	
Motor vehicle	80(53.69%)	37(%)	
Toilet type			<0.05
Sit-down toilet	97(65.10%)	101(%)	
Squat toilet	52(34.90%)	17(%)	
Walk up and down stairs			0.004
Yes/ No	101/48	59/59	
Vegetables and fruits			0.004
Every day	84(56.37%)	79(66.95%)	
Once a week	40(26.85%)	15(12.71%)	
Once a month	14(9.40%)	6(5.08%)	
Hardly	11(7.38%)	18(15.26%)	
Red meat and its products			0.010
Every day	17(11.41%)	24 (20.34%)	
Once a week	66(44.30%)	51(43.22%)	
Once a month	55(36.91%)	26(22.03%)	
Hardly	11(7.38%)	17(14.41%)	
Dairy products			0.003
Every day	40(26.85%)	22(18.64%)	
Once a week	50(33.55%)	37(31.36%)	
Once a month	44(29.53%)	27(22.88%)	
Hardly	15(10.07%)	32(27.12%)	
Anxiety and depression			0.001
Yes/ No	64/85	28/90	

### Risk prediction models

The findings derived from the binary logistic regression analysis are presented in Table 5. The data reveal that the most prominent associations pertained to educational level, with the absence of a diploma demonstrating the strongest linkage, presenting an odds ratio (OR) of 4.474. Following this, notable associations were identified with respect to age, specifically within the 60–69-year-old bracket, yielding an OR of 3.927. The type of toilet facility, specifically the use of squat toilets, was also significantly associated with the condition, with an OR of 2.929. Furthermore, a family history of KOA emerged as a substantial risk factor, with an OR of 2.562. In addition to these factors, the odds ratio for anxiety and depression was determined to be 2.431, indicating a considerable association with the condition under investigation.

**Table 5:** Binary logistic regression model analyzing the association between risk factors and the incidence of KOA.

Risk Factor	Odds Ratio (95% Confidence Interval)	P-value
Age(y)		
40-49	1	0.006
50-59	2.763(1.355,5.636)	0.005
60-69	3.927(1.825,8.450)	<0.05
＞ 70	2.904(1.289,6.544)	0.010
Educational level		
High school or above	1	
No diploma	4.474(1.811,11.053)	0.001
Junior diploma	2.769(1.272,6.028)	0.010
Family history of KOA		
No	1	
Yes	2.562(1.444,4.544)	0.001
Toilet type		
Sit-down toilet	1	
Squat toilet	2.929(1.269,4.140)	0.006
Transportation		
Motor vehicle	1	
Walk	2.265(1.156,3.450)	<0.05
Bike	1.240(0.114,2.507)	<0.05
Anxiety and depression		
No	1	
Yes	2.431(1.456,4.058)	0.001
Dairy products		
Hardly	1	0.001
Every day	0.545(0.289,1.029)	0.061
Once a week	0.600(0.299,1.204)	0.151
Once a month	0.232(0.115,0.466)	<0.05

Note: OR = Odds Ratio; CI = Confidence Interval.

### Validation of the risk prediction models

**Fig 2:**
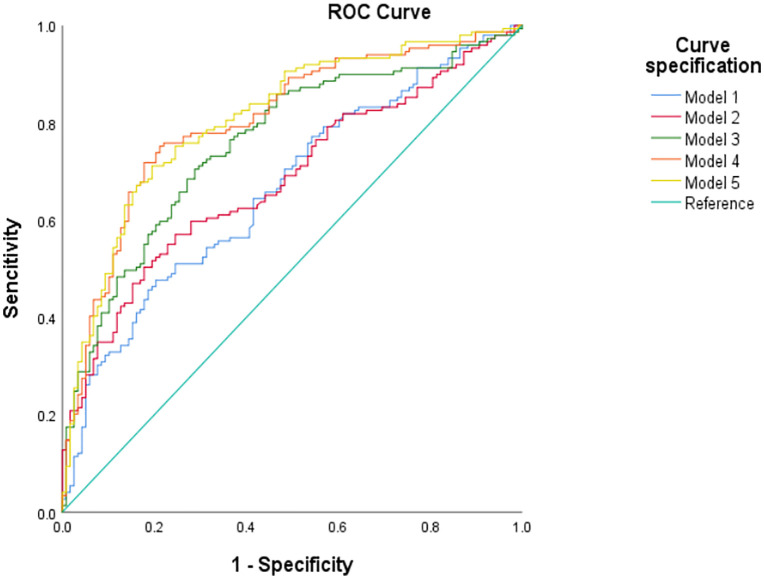
Receiver operating characteristic (ROC) curves comparing the performance of five risk prediction models.

The Hosmer–Lem show χ² statistic for assessing goodness of fit and the Area Under the Receiver Operating Characteristic (AUC) for the distinct risk factor groups across all risk prediction models are presented in Table 6. Notably, the inclusion of lifestyle variables into the model resulted in a significant enhancement of the AUC, increasing from 0.68 to 0.78. Model 5 has the largest AUC value (0.81), and it can be seen from Table 7 that it achieves the best predictive performance. Fig 2 illustrates the ROC curves for the five models, providing a visual representation of their predictive accuracy.

**Table 6:** Validation of the Risk Prediction Models: Calibration and Discrimination.

Model Component(s)	Discrimination: AUC (95% CI)	Calibration: Hosmer–Leme show p Value
Age, gender, weight, height (Model 1)	0.67(0.60 to 0.73)	0.449
Model 1 + Family history of KOA (Model 2)	0.68(0.62 to 0.75)	0.531
Model 2 + Life style (Model 3)	0.78(0,70 to 0.82)	0.367
Model 3) + Dietary habit (Model 4)	0.80(0.75 to 0.86)	0.358
Model 4 + Emotion (Model 5)	0.81(0.76 to 0.86)	0.984

Notes: AUC, area under the curve; Model 1 includes age, gender, weight, and height; Model 2 incorporates Model 1 components plus family history of KOA; Model 3 includes Model 2 components plus lifestyle factors such as toile type and transportation; Model 4 incorporates Model 3 components plus dietary habits, specifically dairy product consumption; Model 5 includes all components from Model 4 with the addition of emotional factors, namely anxiety and depression. The Hosmer–Leme show test was utilized to assess model calibration, with higher p-values indicating better calibration. Discrimination was evaluated using the area under the receiver operating characteristic curve (AUC), with higher AUC values indicating superior model performance.

**Table 7:** Performance Comparison of Classification Models.

Model	Accuracy	Precision	Recall	F1 Score
Model1	0.6217	0.6277	0.7919	0.7003
Model 2	0.6255	0.6433	0.7383	0.6875
Model 3	0.6854	0.7044	0.7517	0.7273
Model 4	0.7105	0.7233	0.7770	0.7492
Model 5	0.8491	0.8467	0.8517	0.8492

## Discussion

The objective of our investigation was to delineate the risk factors and to develop a predictive model for KOA utilizing a questionnaire-based survey approach. As illustrated in Table 3, our analysis revealed that gender, body weight, and age did not exhibit statistically significant differences between the two study groups, a finding that contrasts with the outcomes of prior research. This discrepancy warrants further exploration and contextualization within the existing body of literature. The observed discrepancies in our findings may be attributed primarily to two pivotal factors. Firstly, regional disparities play a significant role, as our study population is situated in the arid climate of northwest China, where the inhabitants predominantly engage in agricultural activities. This unique demographic context may influence the prevalence and manifestation of KOA. Secondly, the limited sample size employed in our research may introduce bias into the statistical outcomes. Despite the lack of statistical significance in age distribution between the two groups, binary logistic regression analysis (refer to Table 5) revealed a notable trend: the incidence of KOA positively correlates with advancing age. This finding underscores the importance of considering age as a critical determinant in the etiology of KOA.

In the present study, significant statistical differences were observed in occupation, educational level, height [12], and BMI between the two groups. Specifically, the KOA cohort exhibited a higher prevalence of individuals engaged in farming, animal husbandry, and fishery compared to the control group. This occupational trend may serve as a potential risk factor for the development of KOA.

Utilizing a binary logistic regression model to examine the association between risk factors and the incidence of KOA, our findings, as presented in Table 5, indicate a notable disparity in KOA prevalence across different age groups. Specifically, individuals within the 60–69-year age bracket were found to be significantly more susceptible to KOA when compared to their 40–49-year-old counterparts.

Furthermore, this investigation elucidated that lifestyle and dietary habits are significant determinants of the incidence of KOA. Notably, our findings revealed that individuals who utilize squat toilets are at a heightened risk for developing KOA compared to those who use sit-down toilets, with an odds ratio (OR) of 2.929. Moreover, we observed that individuals who frequently engage in walking and cycling exhibit a greater propensity to develop KOA than those who predominantly use motor vehicles. These findings underscore the pivotal role of lifestyle choices in the etiology of KOA.

In the context of educational attainment, individuals without a high school diploma (odds ratio [OR] 4.474) or those with a junior high school diploma (OR 2.769) exhibited a significantly elevated risk of KOA compared to those with a high school education or higher. During the questionnaire administration, we observed that individuals with lower educational qualifications tended to experience poorer living conditions. Consequently, their dietary intake was often lacking in essential nutrients, such as vegetables, fruits, dairy products, and proteins. Notably, our findings revealed that dairy consumption served as a protective factor against KOA, particularly when compared to those who rarely consumed dairy products (refer to Table 5).

In the present study, we constructed five predictive models utilizing binary logistic regression to anticipate the onset of KOA. Given that our cohort is in its incipient stages, we confined our validation efforts to internal checks, as detailed in Table 6. Future longitudinal follow-ups will enable us to conduct external validation. As indicated in Table 6, Model 5 exhibited the highest discriminative power, incorporating variables such as age, gender, body weight, height, family history of KOA, lifestyle, dietary habits, and emotional state [13]. Notably, the most substantial enhancement in the model’s predictive capability was achieved with the integration of lifestyle habits and dietary patterns.

## Conclusions

In summation, this study underscores that beyond established risk factors including age, gender, height, and weight, lifestyle and dietary habits emerge as significant contributory elements in the etiology of KOA. These modifiable factors not only serve as potential risk markers but also hold promise as predictive indicators for the onset of KOA, warranting further investigation and the development of targeted interventions.

## Supporting information

S1 File Questionnaire. (PDF)

S1 Table Original data. (XLSX)
