# Supplementary material for: Exploration of risk factors for the incidence of knee osteoarthritis in rural areas of northern China and the establishment of a prediction model
Source: PLoS One. 2025 Dec 29;20(12):e0338003. doi: 10.1371/journal.pone.0338003 (PMC12747343; doi:10.1371/journal.pone.0338003)
Supplement: S1 File — (PDF) [file pone.0338003.s001.pdf]

Epidemiological survey of knee osteoarthritis prevalence in the northern region of China.

Dear participant,

It would be greatly appreciated if you could spare a moment of your time to participate in this questionnaire survey. The purpose of this survey is to understand the epidemiological characteristics of knee osteoarthritis in the northern region of China. The questionnaire will be filled out anonymously, and the results of this research will solely be used for academic purposes. We kindly request that you fill it out truthfully. We are very grateful for your cooperation.

Best regards.

1. Have you had recurrent knee pain in the past 1 month? [Multiple Choice]

○Yes

○No

2. How long does your knee stiffness last in the morning?

○<30 minutes

○>30 minutes

○The joints are not stiff

3. Your name?[Fill-in-the-blank questions]

----

4. Your gender?[Fill-in-the-blank questions]

○Male

○Female

Menarcheal age? [Fill in the blank] *(Depends on the 4rd question, 2nd option)

Has menopause occurred? [Single choice question] *

○ Yes

○ No

Depends on the 4rd question, 2nd option

Menopausal age? [Fill in the blank] *

5. Your identification number?[Fill-in-the-blank questions]

----

6. Your ethnicity?

○Han nationality

○Hui nationality

○Manchu nationality

○Mongolian nationality

○Other ethnic groups / Ethnic minorities

----

7. Please select the province, city, and region: [Fill-in-the-blank question] *

----

8. Height (cm)? [Fill-in-the-blank question] *--------------------------

9. Weight (kg)? [Fill-in-the-blank question] *--------------------------

10. Education Level: [Single Choice] *

○ No formal education

○ Primary School

○ Junior High School

○ High School/Technical Secondary School/Vocational School

○ Bachelor’s Degree or Associate Degree

○ Master’s Degree and above

11. Current Marital Status? [Single Choice] *

○ Unmarried

○ Married

○ Widowhood

○ Divorced

○ Separated

12. What is your current occupation or the occupation you had before retirement? [Single Choice]

○ Farmer, Shepherd, Fisherman

○ Worker

○ Waiter/Waitress or Service Staff

○ Doctor, Journalist, Teacher

○ Self-employed Individual, Corporate or Institutional Manager

○ Government Employee, Civil Servant, General Staff

○ Unemployed Individual

13.If you are not retired but no longer working, what is the main reason? [Single Choice] *

○ Not retired

○ Health issues

○ Working conditions

○ Retirement at the normal age

14.Smoking Status [Single Choice] *

○ Non-smoker

○ Smoker

15.Smoking History [Single Choice] *

○ <1 year

○ 1-10 years

○ 11-20 years

○ 21-30 years

○ 30 years or more

Dependent on the second option of Question 14

16.Daily Smoking Amount (sticks/day) [Single Choice] *

○ Less than 10 sticks

○ 11-20 sticks

○ 21-30 sticks

○ 31-40 sticks

○ More than 40 sticks

Dependent on options 1-5 of Question 16.

17.Smoking Cessation Status [Single Choice] *

○ Not quit smoking

○ Quit smoking

Dependent on options 1-5 of Question 16

18. Smoking Cessation Duration [Single Choice] *

○ 3 months

○ 3-6 months

○ 6-12 months

○ 1 year or more

Dependent on the second option of Question 18

19. Drinking Status [Single Choice] *

○ Non-drinker

○ Drinker

20. Drinking Frequency [Single Choice] *

○ At least once a day

○ At least once a week

○ At least once a month

○ At least once a year

○ Occasionally, less than once a year

Dependent on the second option of Question 19

21. Alcohol Cessation Status [Single Choice] *

○ Quit drinking

○ Not quit drinking

Dependent on options 1-5 of Question 21

22.Alcohol Abstinence Duration [Single Choice]

○ Less than 1 year

○ More than 1 year

23. Fresh vegetables and fruits [Single Choice] *

○ Almost never eat

○ Daily

○ Weekly

○ Monthly

24. Beef and lamb [Single Choice] *

○ Almost never eat

○ Daily

○ Weekly

○ Monthly

25. Pork (for Han nationality only) [Single Choice] *

○ Almost never eat

○ Daily

○ Weekly

○ Monthly

- Depending on Question 5, options 1; 3; 4

26. Fish, shrimp, seafood, etc. [Single Choice] *

○ Almost never eat

○ Daily

○ Weekly

○ Monthly

27. Legumes, dairy products (milk, soy milk, etc.) [Single Choice] *

○ Almost never drink

○ Daily

○ Weekly

○ Monthly

28. Deep-fried foods such as fried dough sticks, fried pancakes, etc. [Single Choice] *

○ Almost never eat

○ Daily

○ Weekly

○ Monthly

29. Do you usually take the stairs? [Single Choice] *

○ Yes

○ No

Number of floors you take the stairs? [Fill-in-the-blank] *

____

- Depending on the first option of Question 29

30. Residential Environment [Single Choice] *

○ Dry Environment

○ Humid Environment (residence is close to ponds, canals, artificial lakes, etc.)

○ Dark Environment (the residence is barely exposed to sunlight)

31. Main Defecation Habit [Single Choice] *

○ Squatting Toilet

○ Sitting Toilet

32. Duration of Each Squatting Defecation [Single Choice] *

depending on option 1 of question 34

○ 5 minutes

○ 6-10 minutes

○ 11-15 minutes

○ 16-20 minutes

○ More than 21 minutes

33. What are the usual heating methods in your home during winter? [Multiple Choice] *

□ No Heating

□ Central Heating (including floor heating, geothermal)

□ Burning Coal, Wood, Charcoal, Straw, etc.

34. Source of Drinking Water for Daily Use? [Multiple Choice] *

□ Bottled Pure Water

□ Tap Water with Water Purifier Installed

□ Tap Water without Water Purifier Installed

□ Well Water, Spring Water

35. What are your usual methods of work or daily travel? [Multiple Choice] *

□ Walking

□ Bicycle and other pedal-powered vehicles

□ Motorcycle, electric vehicle, private car, bus, etc.

36.How many kilometers do you walk every day? [Fill-in-the-Blank] *

____

Dependent on the first option of Question 35

37.How many kilometers do you ride a bicycle every day? [Fill-in-the-Blank] *

____

Dependent on the second option of Question 35

38. Has your knee ever been injured? [Single Choice] *

○ No knee injury

○ Right knee injured

○ Left knee injured

○ Both knees injured

39. Reason for knee injury [Single Choice] *

○ Fracture

○ Sprain

○ Fall

Dependent on the second, third, and fourth options of Question 38

40. Injured site [Multiple Choice] *

□ Meniscus

□ Anterior cruciate ligament

□ Posterior cruciate ligament

□ Medial and lateral collateral ligaments

□ Articular cartilage

□ Femur and tibial plateau

□ Site of injury unclear

Dependent on the first, second, and third options of Question 39

41. Has your knee undergone surgery? [Single Choice] *

○ No surgery

○ Left knee surgery

○ Right knee surgery

○ Both knees surgery

42. Site of surgery [Multiple Choice] *

□ Meniscus repair

□ Meniscus removal

□ Cartilage repair

□ Anterior cruciate ligament reconstruction

□ Posterior cruciate ligament reconstruction

□ Distal femur, tibial plateau

□ Knee replacement surgery

Dependent on the second, third, and fourth options of Question 44

43. What are your usual exercise methods? [Multiple Choice] *

□ Walking

□ Running

□ Square dance

□ Ball sports such as basketball, football, badminton, etc.

□ Mountaineering

□ Swimming

□ Fitness, aerobics, yoga, etc.

□ Professional athlete

44. Frequency of Regular Exercise [Single Choice] *

○ Rarely exercise

○ Daily

○ Weekly

○ Monthly

45. Duration of Each Exercise Session [Single Choice] *

○ Less than 30 minutes

○ 30-60 minutes

○ 60-120 minutes

○ 2-3 hours

○ 3-5 hours

○ More than 5 hours

46. Do you suffer from hypertension? [Single Choice] *

○ Yes

○ No

Dependent on Question 49, Option 1

47. Maximum Systolic Blood Pressure? [Single Choice] *

○ 140-159

○ 160-179

○ ≥180

Dependent on the first option of Question 46

48. Maximum Diastolic Blood Pressure? [Single Choice] *

○ 90-99

○ 100-109

○ ≥110

Dependent on the options 1, 2, and 3 of Question 47

49. Oral Antihypertensive Medications [Multiple Choice] *

□ Calcium Channel Blockers (e.g., Nifedipine Slow-Release (Controlled-Release) Tablets)

□ Beta-Blockers (e.g., Metoprolol)

□ ACE Inhibitors (e.g., Captopril)

□ ARBs (e.g., Candesartan Cilexetil Tablets)

□ Diuretics (e.g., Spironolactone)

□ Other Medications

□ Can't Remember the Name of the Medication

□ Not Taking Medication

Dependent on the options 1, 2, 3, 4, 5, 6, and 7 of Question 48

50. Are You Taking Medication Regularly? [Single Choice] *

○ Yes

○ No

Dependent on the options 1, 2, 3, 4, 5, 6, and 7 of Question49

51. Do you have diabetes? [Single Choice] *

○ Yes

○ No

52. Are you taking hypoglycemic drugs? [Multiple Choice] *

□ Metformin

□ Insulin

□ Other

□ Can't remember the name of the drug

□ Not taking any medication

Depends on the first option of Question 51

53. Are you taking medication regularly? [Single Choice] *

○ Yes

○ No

54. Do you have coronary heart disease? [Single Choice] *

○ Yes

○ No

55. Do you have hyperlipidemia? [Single Choice] *

○ Yes

○ No

56. Hyperlipidemia treatment drugs [Multiple Choice] *

□ Statins

□ Fibrates

□ Other medications

□ Can't remember the name of the drug

□ Not taking any medication

Depends on the first option of Question 55

57. Are you taking medication regularly? [Single Choice] *

○ Yes

○ No

58. Do you have rheumatoid arthritis? [Single Choice] *

○ Yes

○ No

59. Do you have osteoporosis? [Single Choice] *

○ Yes

○ No

60. Have your parents or siblings been diagnosed with knee osteoarthritis? [Single Choice] *

○ Yes

○ No

○ Have knee pain or difficulty walking, but not visited a doctor

61. Is it difficult for you to bend down to pick up things? [Single Choice] *

○ No difficulty

○ Slight difficulty, accompanied by knee discomfort

○ Moderate difficulty, requiring effort to pick up

○ Severe difficulty, need support to pick up

○ Unable to perform by myself

62. Is your daily life (such as work, study, household chores, family, or leisure activities) affected? [Single Choice] *

○ No difficulty

○ Slight difficulty, accompanied by knee discomfort

○ Moderate difficulty, requiring effort

○ Severe difficulty, hard to complete

○ Unable to perform by myself

63. Do you have knee pain, tenderness, swelling, or stiffness? [Multiple Choice] *

□ Pain

□ Swelling

□ Tenderness

□ Stiffness

□ No symptoms mentioned above

64. Are you usually anxious or depressed? [Single Choice] *

○ No anxiety or depression

○ Slight anxiety or depression

○ Moderate anxiety or depression

○ Severe anxiety or depression

○ Very severe anxiety or depression

65. How severe do you think your knee pain is? [Single Choice] *

○ No pain

○ Mild pain, bearable, normal life and sleep

○ Bearable, normal life and sleep

○ Moderate pain

○ Slightly affects sleep, needs painkillers

○ Severe pain

○ Affects sleep, needs anesthetic painkillers

○ Severe pain

○ Greatly affects sleep, with other symptoms

○ Unbearable

○ Greatly affects sleep, with other symptoms

66. Do you have an X-ray examination of the knee? [Single Choice] *

○ Yes

○ No

67. Do you have an MRI examination of the knee? [Single Choice] *

○ Yes

○ No

68. Have you had a blood test today? [Single Choice] *

○ Yes

○ No
